# Development of a measurement of doctor-patient communication quality scale

**DOI:** 10.3389/fpubh.2025.1606403

**Published:** 2025-08-11

**Authors:** Jiayi Shao, Minhui Wen, Yuqing Zhang, Liping Zhang, Jiangjie Sun

**Affiliations:** ^1^School of Health Care Management, Anhui Medical University, Hefei, China; ^2^School of Marxism, Anhui Medical University, Hefei, China; ^3^School of Management, Hefei University of Technology, Hefei, China

**Keywords:** doctor-patient communication, doctor-patient relationship, communication quality, measurement, scale

## Abstract

**Background:**

The number of medical disputes in China has been increasing, highlighting the urgent need to improve doctor-patient relationships.

**Objective:**

To improve doctor-patient relationships and enhance communication, a scale for measuring doctor-patient communication quality (DPCQ) was developed.

**Methods:**

Based on existing relevant foreign scales, questionnaire items were designed to collect patients' perceptions of doctor-patient communication quality. Outpatients from a selected hospital were chosen as the study population, and 300 questionnaires were randomly sampled. SPSS 26 was used for preliminary item screening through dispersion trend analysis, *t*-tests, and Pearson correlation analysis. Exploratory factor analysis (EFA) was conducted to assess the reliability of the questionnaire. Structural validity was examined using structural equation modeling (SEM) in R 4.4.3. Convergent and discriminant validity were evaluated using average variance extracted (AVE), composite reliability (CR), and maximum shared variance (MSV).

**Results:**

The EFA identified two factors: Factor 1 (Interactive Communication and Emotional Support) and Factor 2 (Health Education and Behavioral Guidance), with a cumulative variance contribution rate of 63.81%. The results of the CFA indicated an acceptable model fit: χ^2^/d*f* = 3.57, AGFI = 0.837, RMSEA = 0.093, CFI = 0.945, NFI = 0.926, PGFI = 0.609, and PNFI = 0.733. The final version of the questionnaire demonstrated excellent internal consistency, with a Cronbach's α coefficient of 0.950. In addition, AVE exceeded the threshold of 0.50, CR values were greater than 0.70, and MSV for each factor was lower than its corresponding AVE, supporting both convergent and discriminant validity of the scale.

**Conclusion:**

The scale for measuring doctor-patient communication quality demonstrates good reliability and validity, making it a suitable measurement tool for research on doctor-patient communication.

## 1 Introduction

In recent years, the tension in doctor-patient relationships has become a widespread modern issue, not only in China but also globally (1, 2). In China, a series of factors, such as market-oriented healthcare reforms (3), have contributed to the frequent occurrence of medical violence and incidents of harm against doctors (4, 5). Patients are increasingly aware of their rights and have higher demands regarding the service attitude, quality, and efficiency of healthcare institutions. The doctor-patient relationship is commonly defined as “a form of communication expressed through interaction between doctors and patients” (6). The quality of the entire medical process, including accurate diagnosis and effective treatment of diseases, largely depends on the quality of the doctor-patient relationship (7). Today, patients have access to a vast amount of medical information through new media, leading them to take favorable treatment outcomes for granted. However, patients often lack a clear understanding of the uncertainties, risks, and individual variability involved in many medical technologies (8). Research has shown that good doctor-patient communication can improve patients' emotions and enhance treatment outcomes (9). Many studies have also emphasized the reciprocity of doctor-patient communication, gradually focusing on patients' perceptions of the communication (10). Therefore, this study conducts a measurement of doctor-patient communication quality from the patient's perspective, aiming to improve doctor-patient communication quality, strengthen the doctor-patient relationship, and promote better medical outcomes.

## 2 Literature review

The doctor-patient relationship is a matter of global concern. Studies from both domestic and international contexts have shown that doctor-patient trust, communication, quality of medical services, and patient satisfaction are key factors influencing the state of this relationship. Ha and Longnecker (11) conducted a comprehensive analysis of doctor-patient communication, highlighting five critical elements for improvement: the application of communication skills, updates in communication training, bidirectional information exchange between doctors and patients, appropriate management of doctor-patient conflict, and consideration of health-related beliefs and values. Numerous studies have explored how doctor-patient communication functions throughout the diagnostic and therapeutic process. As one of the fundamental driving forces in healthcare delivery, doctor-patient communication affects not only the treatment process but also the patient's adherence to medical advice (12). Street (13) suggested that certain communication strategies could directly or indirectly influence patient health outcomes. Wang et al. (14) found that trust in doctors is indirectly influenced by communication, particularly because patients often lack sufficient psychological preparation due to their relatively low risk perception in medical contexts (15). Therefore, doctor-patient trust may mediate the relationship between communication and patients' perception of medical risk. Other researchers have proposed that expected treatment outcomes and patient-centered care can positively affect communication satisfaction (16). When physicians actively attempt to understand the patient's perspective—including background, beliefs, and opinions—and are able to share that understanding, communication effectiveness is significantly enhanced (17, 18). In summary, doctor-patient communication plays a crucial role in the medical process, shaping patient trust, health perceptions, and therapeutic outcomes.

High-quality doctor-patient communication can significantly improve patient satisfaction and enhance the loyalty of patients and their families toward the hospital, bringing additional social and direct economic benefits to the hospital. Therefore, in recent years, scholars both domestically and internationally have conducted extensive research on how to reflect the quality of doctor-patient communication, accumulating a wealth of research findings. Matusitz and Spear (12) pointed out that doctor-patient communication is a powerful indicator of healthcare service quality and can be used to assess patients' self-management behaviors and health status. Scales measuring doctor-patient communication quality from the patient's perspective include scales for evaluating doctors' communication behaviors, such as the Interpersonal Relationship Process Scale (19, 20), the Empathy Scale for consultations (21), and the Patient Involvement in Decision-Making Evaluation Scale (22). Berkenstadt et al. developed the Personal Control Perception Scale (23), which is administered before and after the consultation. The differences between the two surveys are used to assess the impact of the consultation on the patient's sense of psychological control and the extent of that impact. If a patient actively participates in decision-making during doctor-patient communication, the Decision Conflict Scale (24) can quantify the patient's uncertainty in decision-making and identify the factors that cause uncertainty during the deliberation and subsequent choices, along with the Decision Satisfaction Scale (25). Research on patient satisfaction in China began later than in other countries, with scholars starting to explore this area only in the late 20th century. Representative domestic studies include: Zhang et al. (26), who developed a comprehensive hospital emergency patient satisfaction scale with 26 items across 8 factors, such as treatment outcomes and costs, doctor services, and the medical environment. Yan et al. (27), through follow-up visits to discharged patients, established a satisfaction evaluation system with 25 items across 8 aspects, including service attitude, medical costs, and medical ethics (28). In summary, although multiple tools have been developed to measure doctor-patient communication, several limitations remain. First, many existing scales focus on a single aspect of communication, such as physician empathy or patient involvement in decision-making, and therefore fail to capture the multidimensional nature of doctor-patient communication comprehensively. Second, many instruments are directly adapted from foreign contexts, lacking sufficient cultural and systemic alignment with the Chinese healthcare environment, which compromises their measurement accuracy and interpretability. Third, some scales were developed without adequately incorporating the patient's perspective, overlooking the subjective experiences of patients during the communication process.

Based on the above, this study aims to develop a doctor-patient communication quality scale suitable for the Chinese context, in order to address the limitations of existing tools. The DPCQ is designed to reflect patients' subjective experiences while ensuring high content validity and structural validity, thus providing a foundational instrument for future research and interventions in the field of doctor-patient communication quality.

## 3 Research methods

### 3.1 Item design

The purposes of a patient's visit can be summarized as: understanding health status, receiving information about diseases and related health education, making the best decisions, and gaining the doctor's support and assistance in coping with the disease. The communication outcomes include: alleviating psychological stress, increasing communication satisfaction, reducing the uncertainty of treatment plans, improving quality of life, and enhancing a sense of personal control. Achieving these visit purposes not only represents the outcome quality of doctor-patient communication but also indirectly reflects the quality of the communication process, as well as the communication behaviors of both parties, especially the doctor's communication behavior. The selection of items followed several criteria. First, priority was given to items directly related to the quality of doctor-patient communication, ensuring the inclusion of aspects such as information exchange, emotional support, and communication skills. Second, items were referenced from scales with established reliability and validity in both domestic and international studies, to guarantee scientific rigor and reliability. Additionally, cultural adaptability was fully considered to ensure that the content could be accurately understood and accepted by Chinese patient populations.

Based on this logic, relevant foreign scales were integrated and their content was refined and translated, resulting in a draft of a 30-item questionnaire. This includes the following scales:

The Interpersonal Process of Care Scale (IPC) (19, 20) is designed to assess the quality of interpersonal care across different patient groups. It presents and validates a conceptual framework for distinguishing specific components of the interpersonal process from the patient's perspective, aiming to identify specific aspects of the interpersonal relationship that may serve as targets for quality assessment and improvement. The Consultation and Relational Empathy Scale (CARE) (21) is generally used as a tool to measure patients' perceptions of relational empathy during medical consultations. The Observing Patient Involvement in Decision Making Scale (OPTIONG) (22) measures the behaviors of doctors in creating opportunities for patient involvement in decision-making during doctor-patient communication. It evaluates the extent to which clinicians involve patients in decisions under various circumstances (excluding emergencies or other adverse situations). The Personal Perceived Control Scale (PPC) (23) is used to assess the impact of the consultation on the patient's sense of psychological control and the degree of that impact. The Satisfaction with Decision Scale (SWD) (25) evaluates patients' satisfaction with the decision-making process in doctor-patient communication.

After conducting a pilot study with 150 patients, we performed preliminary item analysis and exploratory factor analysis (EFA) to assess the quality of the items. Based on the results, some items with inadequate performance were removed (see Table 1). Table 1 presents the survey items and their sources used in the pilot study, clearly indicating which items were retained (marked as T1–T16) and which were excluded. Following this screening process, a preliminary doctor-patient communication quality measurement scale consisting of 16 items was developed. Using a five-point Likert scale with positive wording, each item has five options: “Strongly Disagree,” “Disagree,” “Neutral,” “Agree,” and “Strongly Agree.” A score of 1–5 is assigned to each item, with higher scores indicating better doctor-patient communication quality.

**Table 1:** Survey items tested in the pilot study and their selection status for the final instrument.

**Item**	**Content**	**Source scale**	**Inclusion**
–	Doctors often use medical terms you don't understand	Adapted from IPC	×
–	You often fail to understand the doctor due to rapid speech	Adapted from IPC	×
–	Doctors ensure you understand your health condition	Adapted from IPC	×
T1	The doctor explains the purpose and necessity of the examination to me	Adapted from IPC	√
T2	If the doctor does not explain clearly and just asks me to follow instructions, I feel confused about the diagnosis and treatment process	Adapted from IPC	√
T3	The doctor advises me to monitor symptom changes to seek timely medical attention	Adapted from IPC	√
T4	Following the doctor's treatment plan can improve my health condition	Adapted from IPC	√
–	You feel pressured by the doctor to accept uncomfortable treatment	Adapted from IPC	×
T5	The diet and lifestyle recommended by the doctor contribute to my recovery	Adapted from IPC	√
T6	The doctor asks about any difficulties I encounter while receiving treatment	Adapted from IPC	√
–	Doctors respect your privacy during examinations or questions	Adapted from IPC	×
–	You often feel discriminated by doctors due to education or income	Adapted from IPC	×
T7	The doctor's treatment makes me less concerned about my health condition	Adapted from PPC	√
–	Doctors praise you for taking good care of yourself	Adapted from IPC	×
T8	The doctor has a warm and friendly attitude toward me	Adapted from CARE	√
–	Doctors give you time to describe your condition in detail	Adapted from CARE	×
T9	The doctor listens carefully to what I have to say	Adapted from CARE	√
T10	The doctor cares about the details of my life	Adapted from CARE	√
–	Doctors show empathy and connect with you on a human level	Adapted from CARE	×
T11	The doctor provides active treatment and honestly informs me about my condition	Adapted from CARE	√
T12	The doctor answers my questions thoroughly and clearly	Adapted from CARE	√
–	Doctors help you manage your illness by discussing self-care options	Adapted from CARE	×
–	Doctors involve you in action planning and decision-making	Adapted from CARE	×
T13	The doctor carefully considers my condition when diagnosing it	Adapted from OPTIONG	√
–	Doctors include “no treatment” as an option when appropriate	Adapted from OPTIONG	×
T14	The doctor informs me about the various treatment options for my condition	Adapted from PPC	√
T15	The doctor explains the advantages and disadvantages of different treatment options	Adapted from OPTIONG	√
–	Doctors ask whether you prefer paper or electronic results	Adapted from OPTIONG	×
T16 –	The doctor takes my opinions into account when making medical decisions	Adapted from SWD	√
	Doctors give you time to make medical decisions	Adapted from OPTIONG	×

IPC, Interpersonal Process of Care scale (19, 20); CARE, Consultation and Relational Empathy scale (21); OPTING, Observing Patient Involvement in Decision Making scale (22); PPC, Personal Perceived Control scale (23); SWD, Satisfaction with Decision scale (25). Items were removed based on the results of preliminary item analysis and exploratory factor analysis (EFA) conducted during the pilot study.

### 3.2 Data collection

In this study, a random sampling method was employed to distribute questionnaires to adult outpatients aged 18 years and above in the outpatient departments of three hospitals in Anhui Province. The inclusion criteria were outpatients with basic reading ability who provided informed consent and were willing to complete the questionnaire voluntarily. Patients with mental disorders or those with hearing or speech impairments were excluded. Participants were allowed to ask questions if they had any concerns, and the questionnaire took approximately five minutes to complete (29, 30). A database was established using EpiData 3.1 software to input and clean the data. A total of 150 valid questionnaires were collected for the pilot study. According to the requirement for an appropriate sample size, with 10–20 samples per item (31), 300 valid data sets were collected in the formal data collection phase.

### 3.3 Analysis tools

**Figure 1:**
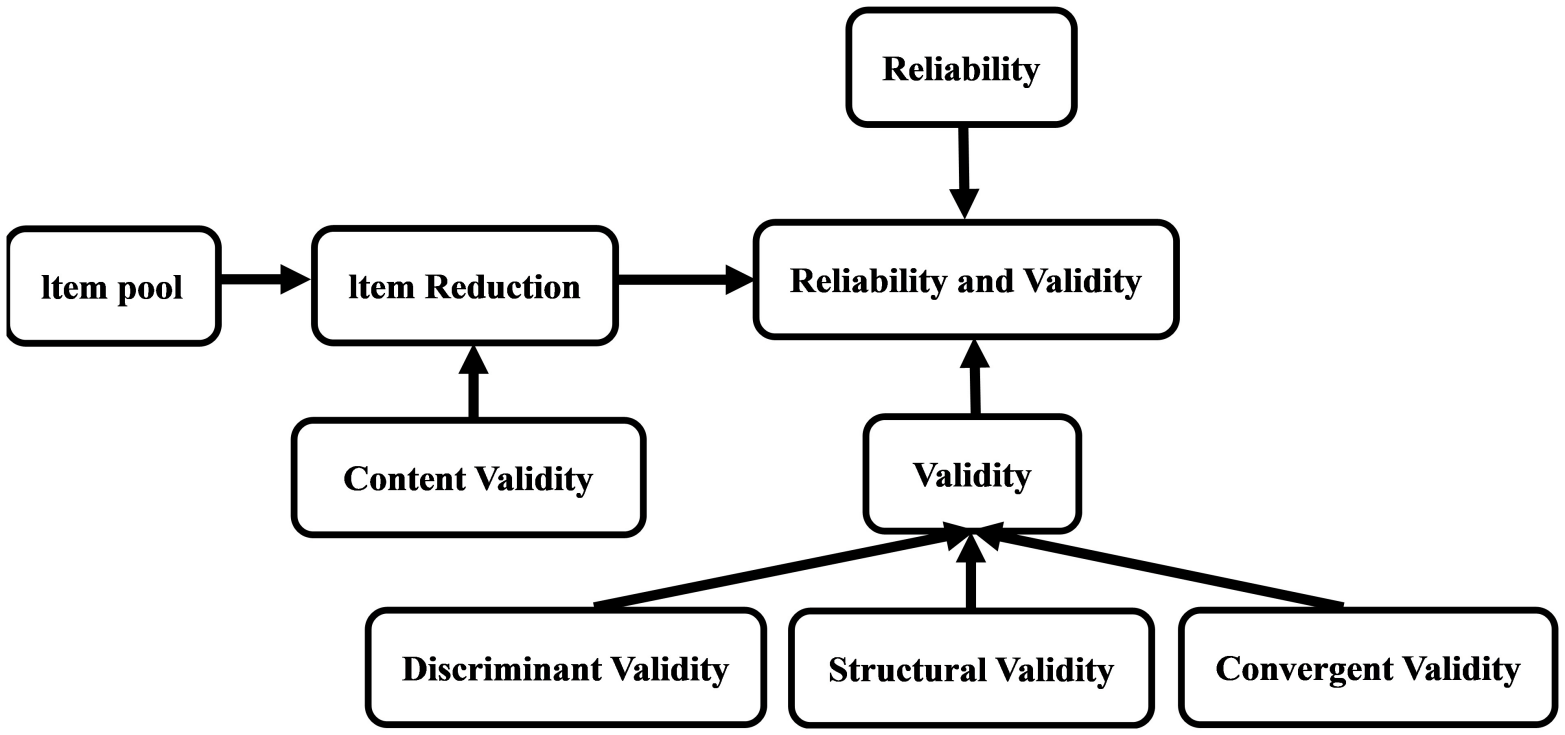
Research roadmap.

The data analysis for this study was conducted using SPSS 26 and R 4.4.3 software. SPSS 26 was used to assess the distribution characteristics of the data, inter-group differences, relationships between variables, scale reliability, and latent structures. R software was used to construct structural models to examine the structural validity of the DPCQ. Discriminant validity and convergent validity were evaluated using AVE, CR, and MSV to verify whether the DPCQ accurately measures predefined concepts and distinguishes different concepts. Through these analysis methods, the reliability and validity of the DPCQ were verified to ensure the reliability of the results. The specific research roadmap (32) is shown in Figure 1.

## 4 Results

### 4.1 Demographic characteristics

A total of 327 questionnaires were collected, among which 300 were valid, yielding a response rate of 91.7%. Among the respondents, 91 were male and 209 were female, with a mean age of 39.89 years (SD = 13.72). Other demographic characteristics are presented in Table 2.

**Table 2:** Characteristics of the participants (*n* = 300).

**Variable**	**Category**	***n***	**%**
Gender	Male	91	30.3
	Female	209	69.7
Ethnicity	Han	295	98.3
	Other	5	1.7
Age	18–30 years	96	32.0
	31–50 years	139	46.3
	51 years and above	65	21.7
Educational level	High school or below	102	34.0
	Junior college	88	29.3
	Bachelor's degree	89	29.7
	Postgraduate degree	21	7.0
Religious belief	No	254	84.7
	Yes	46	15.3
Marital status	Unmarried	92	30.7
	Married (currently with a spouse)	197	65.7
	Other	11	3.7
Number of children	None	106	35.3
	One	158	52.7
	Two	36	12.0
Place of residence	Urban	216	72.0
	Town	67	22.3
	Rural	17	5.7
Occupation	Other	198	66.0
	Enterprise/institution employee	90	30.0
	Medical staff	8	2.7
	Civil servant	4	1.3
Monthly household income per capita	≤ 2,000 RMB	7	2.3
	2,001–5,000 RMB	85	28.3
	5,001–8,000 RMB	100	33.3
	8,001–10,000 RMB	53	17.7
	≥10,000 RMB	55	18.3

### 4.2 Discrete trend analysis

The standard deviation was used to reflect the dispersion trend of each item. Generally, when the standard deviation is less than 0.8, the discrimination is considered poor (33). Considering that discrete trend analysis is only the first step in item quality screening and that multiple subsequent rounds of quality assessments will be conducted, we appropriately lowered the screening threshold to 0.7 in this round to reduce the risk of mistakenly excluding items due to overly strict criteria at the preliminary stage. According to the subsequent analysis, the results in Table 3 generally meet the requirements, and no items were deleted.

**Table 3:** Results of the discrete trend method analysis (*n* = 300).

**Items**	**Mean**	**SD**	**Items**	**Mean**	**SD**
T1	3.85	0.923	T9	3.47	1.071
T2	3.75	1.032	T10	3.47	0.962
T3	4.21	0.781	T11	4.06	0.797
T4	4.14	0.763	T12	3.87	0.914
T5	4.15	0.728	T13	3.80	0.897
T6	4.00	0.888	T14	3.85	0.899
T7	3.95	0.789	T15	3.80	0.897
T8	3.84	0.899	T16	3.87	0.886

### 4.3 *T*-test

The total scores of doctor-patient communication quality were ranked from high to low. The top 27% of scores were classified as the high-score group, and the bottom 27% as the low-score group. In this study, the 81 participants with scores ≥68.73 were categorized as the high-score group, and the 81 participants with scores ≤ 57 were classified as the low-score group. The average score for each item in both groups was calculated, and a difference test was conducted between the means of the two groups. Items with a critical ratio (CR) <3.0 or no statistically significant difference (*p* > 0.05) were removed (34). According to the CR value of 9.643 and *p* < 0.05, no items were deleted. Details are shown in Table 4.

**Table 4:** Results of the *t*-test analysis (*n* = 300).

**Items**	***t***	***P***	**Items**	***t***	***P***
T1	−9.298	<0.001	T9	−5.066	<0.001
T2	−5.441	<0.001	T10	−15.854	<0.001
T3	−14.664	<0.001	T11	−19.626	<0.001
T4	−16.153	<0.001	T12	−18.687	<0.001
T5	−16.284	<0.001	T13	−19.128	<0.001
T6	−19.208	<0.001	T14	−19.760	<0.001
T7	−16.128	<0.001	T15	−20.335	<0.001
T8	−20.956	<0.001	T16	−16.305	<0.001

### 4.4 Pearson correlation coefficient method

Pearson correlation analysis was used to examine the relationship between each item and the total score of the initial questionnaire. Items with *r* < 0.4 were removed (35). According to the results in Table 5, items 2 and 9 were deleted.

**Table 5:** Results of the Pearson's correlation coefficient analysis (*n* = 300).

**Items**	***r***	**Items**	***r***
T1	0.575^**^	T9	0.359^**^
T2	0.343^**^	T10	0.746^**^
T3	0.669^**^	T11	0.850^**^
T4	0.748^**^	T12	0.824^**^
T5	0.764^**^	T13	0.836^**^
T6	0.807^**^	T14	0.802^**^
T7	0.782^**^	T15	0.783^**^
T8	0.846^**^	T16	0.805^**^

^**^P < 0.01.

### 4.5 Cronbach's α coefficient method

Cronbach's α coefficient was used to measure the degree of association among items, reflecting internal consistency. Generally, the Cronbach's α coefficient is positively correlated with the number of items in a questionnaire. If removing an item increases the overall Cronbach's α coefficient, it indicates that the item is not consistent with the intended content of the scale and should be deleted (36). As shown in Table 6, the Cronbach's α coefficient for the 16-item scale was 0.934. Therefore, items 2 and 9 were removed.

**Table 6:** Results of internal consistency analysis (*n* = 300).

**Items**	**Cronbach's α after item deletion**	**Items**	**Cronbach's α after item deletion**
T1	0.933	T9	0.941
T2	0.941	T10	0.928
T3	0.930	T11	0.926
T4	0.928	T12	0.926
T5	0.928	T13	0.926
T6	0.926	T14	0.927
T7	0.927	T15	0.927
T8	0.925	T16	0.927

### 4.6 Exploratory factor analysis (EFA)

A Kaiser-Meyer-Olkin (KMO) test and Bartlett's test of sphericity were conducted on the 16 items. The KMO value was 0.952, and Bartlett's test yielded a chi-square value that reached statistical significance (*p* < 0.001), indicating that the data were suitable for EFA. In this study, principal component analysis (PCA) was employed for item selection and extraction, using the orthogonal rotation method (Varimax). Factors with eigenvalues greater than 1 were extracted, and the retention or removal of items was determined based on the highest factor loading and professional judgment. The criteria for item retention and deletion were as follows (32):

1. Items with a maximum factor loading <0.4 were removed.
2. Items that loaded onto two or more factors, with a difference of <0.2 between the highest and second-highest factor loadings, were removed.
3. If a common factor contained fewer than three items, the factor and its corresponding items were removed.

**Figure 2:**
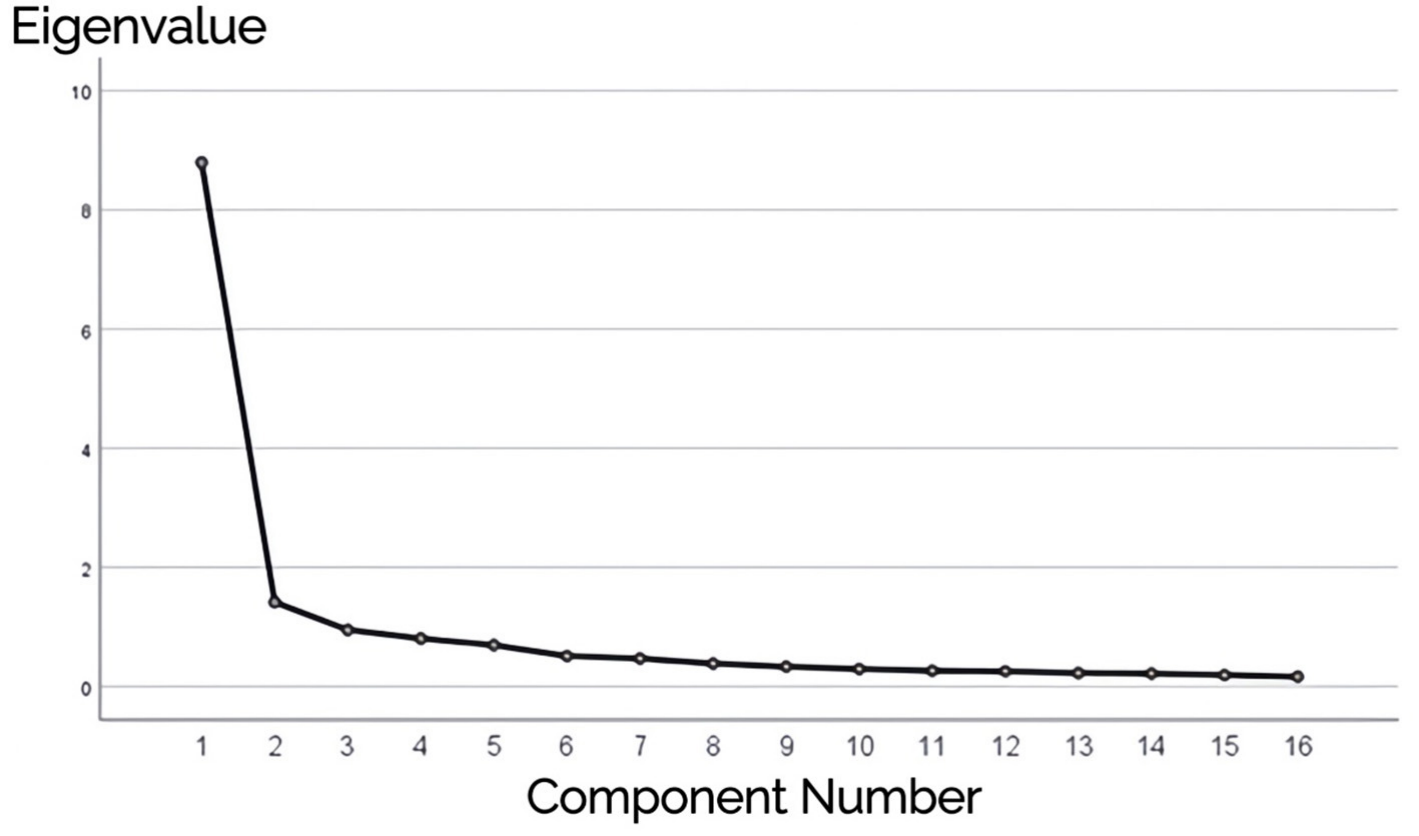
Scree plot.

Further data analysis generated the rotated component matrix, identifying two common factors with eigenvalues >1 (Table 7 and Figure 2). The cumulative variance contribution rate was 63.810%, exceeding 60%. Based on these findings, Item 9 was removed.

**Table 7:** Component matrix after rotation (*n* = 300).

**Items**	**Components**	
	**1**	**2**
Item 13	0.853	
Item 10	0.832	
Item 8	0.828	
Item 12	0.824	
Item 14	0.809	
Item 15	0.805	
Item 16	0.771	
Item 11	0.719	
Item 6	0.662	
Item 7	0.576	
Item 4	0.399	0.759
Item 3	0.291	0.758
Item 5	0.459	0.705
Item 2	−0.096	0.650
Item 1	0.304	0.541
Item 9	0.188	0.251

As can be seen in Figure 2, the eigenvalue of the first factor is significantly higher than those of the other factors, and the decline in eigenvalues after the second factor becomes very gradual. This suggests that the data structure is likely a two-factor model. Based on the above analysis, Item 2 and Item 9 were excluded, and the 14 items were divided into two factors: F1 (Doctor's Interactive Communication and Emotional Support) and F2 (Doctor's Health Education and Behavioral Guidance). F1 includes Items 13, 10, 8, 12, 14, 15, 16, 11, 6, and 7; F2 includes Items 4, 3, 5, and 1.

### 4.7 Construct validity

**Figure 3:**
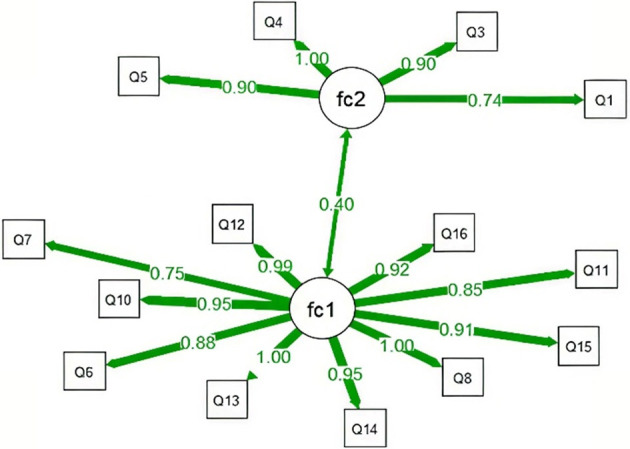
Initial model fit diagram.

The SEM analysis assumes two latent factors, factor 1 and factor 2, with each factor composed of several measurement items (scale items), and there may be correlations between these two factors. Using the lavaan package, the relationships between latent variables and their observed indicators were specified. The initial results of the confirmatory factor analysis (CFA) showed: χ^2^/d*f* = 4.274, AGFI = 0.809, GFI = 0.861, SRMR = 0.056, RMSEA = 0.104, TLI = 0.912, CFI = 0.927, IFI = 0.927, NFI = 0.907, PGFI = 0.624, and PNFI = 0.757. As the RMSEA exceeded 0.10, the model did not meet the ideal fit criteria (see Figure 3).

**Figure 4:**
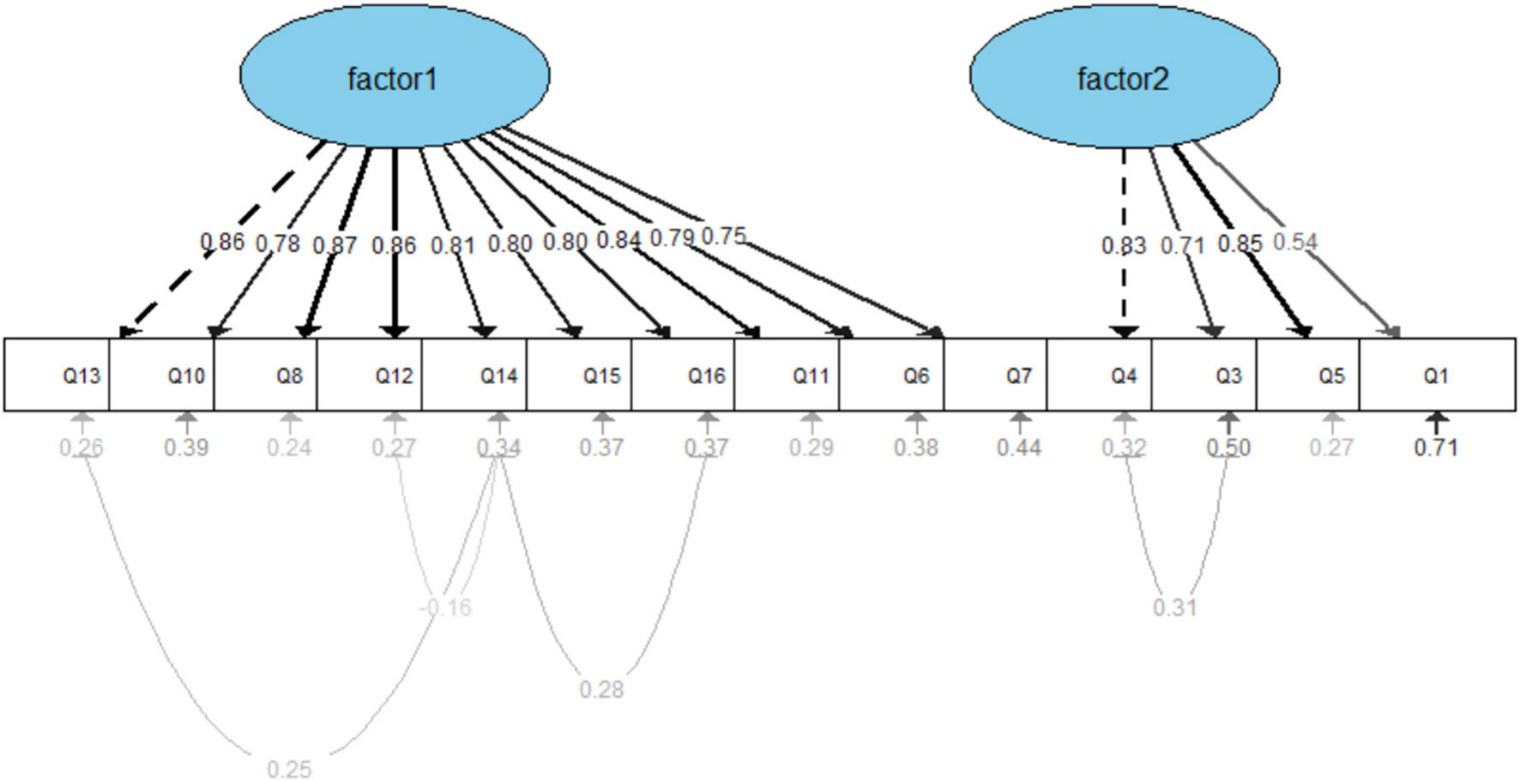
Modified model fit diagram.

Therefore, based on the Modification Indices (MIs) and supported by theoretical justification, several covariance paths were added between measurement error terms. These modifications primarily involved correlating the residuals of items with similar content or expressions, such as Q4 and Q3, Q14 and Q16, among others. The modified model yielded improved fit indices: Absolute fit indices: χ^2^/d*f* = 3.57, AGFI = 0.837, GFI = 0.888, SRMR = 0.049, RMSEA = 0.093; Incremental fit indices: TLI = 0.931, CFI = 0.945, IFI = 0.946, NFI = 0.926; Parsimony fit indices: PGFI = 0.609, PNFI = 0.733. All indices met the acceptable thresholds for model fit, indicating that the structural model of the questionnaire demonstrated a good overall fit (Figure 4).

### 4.8 Discriminant validity and convergent validity

The standard factor loadings of each item were obtained through the CFA (Table 8), with the standard factor loadings being greater than or close to 0.6, indicating a reasonable relationship between each item and its respective factor.

**Table 8:** Coefficients for factor loading (*n* = 300).

**Factors**	**Items**	**Non-standard loading factors (Coef.)**	**Standard error (Std. Err)**	***Z***	***P***	**Standard loading factors (Std. Estimate)**
Factor 1	T13	1.000		–	–	0.862
	T10	0.971	0.057	16.912	0.000	0.780
	T8	1.014	0.049	20.596	0.000	0.872
	T12	1.014	0.051	19.911	0.000	0.857
	T14	0.938	0.046	20.601	0.000	0.811
	T15	0.924	0.053	17.474	0.000	0.796
	T16	0.913	0.052	17.487	0.000	0.797
	T11	0.866	0.045	19.199	0.000	0.840
	T6	0.903	0.053	17.088	0.000	0.785
	T7	0.764	0.048	15.821	0.000	0.748
Factor 2	T4	1.000		–	–	0.825
	T3	0.874	0.057	15.386	0.000	0.705
	T5	0.985	0.063	15.643	0.000	0.853
	T1	0.791	0.085	9.321	0.000	0.539

To further analyze the convergent and discriminant validity of the questionnaire, the standardized coefficients were used to calculate MSV, which was found to be 0.661. AVE for Factor 1 and Factor 2 were 0.872 and 0.840, respectively. CR for Factor 1 was 0.949, and for Factor 2, it was 0.791. Since AVE is greater than 0.5 and CR exceeds 0.7, the questionnaire demonstrates good convergent validity. Additionally, as MSV is smaller than the corresponding AVE, this further indicates that the questionnaire exhibits good discriminant validity.

## 5 Discussion

The results of the dispersion trend analysis and *t*-test indicated that the initial items of the questionnaire were reasonable. Pearson correlation analysis further demonstrated that all 16 items were significantly correlated with the total score, indicating good item-total consistency. This method provides higher item precision and contributes to the optimization of the questionnaire item pool. The results of the EFA showed that Item 2 and Item 9 did not meet the requirements for the factor structure of the DPCQ, thus optimizing the initial scale from a statistical perspective and improving its quality. The overall Cronbach's α coefficient of the DPCQ was 0.950, which is >0.8. The Cronbach's α coefficients for the two factors were 0.95 and 0.82, both greater than 0.7. This indicates that the DPCQ has good reliability, and the internal consistency of each factor is acceptable (37), reflecting the rigor and scientific nature of the research process.

In the CFA, all fit indices of the modified model performed well, indicating that the model has high effectiveness in terms of fit and explanatory power. Both the Absolute Fit Indices and Incremental Fit Indices met the research requirements, suggesting that the model fits the data accurately. Additionally, the values of the Parsimony Fit Indices support the simplicity of the model, demonstrating a good balance between complexity and explanatory power. Further analysis of convergent and discriminant validity revealed that the AVE and CR values for Factor 1 and Factor 2 met the standards, indicating good convergent validity. The MSV value, calculated from the standardized coefficients, shows the associations between the factors, confirming good discriminant validity. In summary, the model excels in terms of fit, convergent validity, and discriminant validity, indicating that the measurement tool in this study has good reliability and validity and demonstrates strong adaptability within the theoretical framework (38). Due to research resource limitations, the same dataset was used for both EFA and CFA. Although best practices recommend conducting EFA and CFA on different samples to avoid result dependence, considering the moderate sample size in this study (*N* = 300) and the fact that only minor adjustments were made to a few items after EFA, the results of the CFA still hold certain reference value. In future research, further exploration of potential improvements to the model can be conducted, such as optimizing measurement variables, expanding the sample size, or adjusting model paths to enhance the fit. Additionally, the stability and applicability of the model should be validated, particularly in terms of its performance across different samples and contexts.

The dimensions and item contents of the questionnaire used in this study are presented below (Table 9).

**Table 9:** Dimensions and items of the DPCQ.

**Factors**	**Items**
F1	13, The doctor carefully considers my condition when diagnosing it
	10, The doctor cares about the details of my life
	8, The doctor has a warm and friendly attitude toward me
	12, The doctor answers my questions thoroughly and clearly
	14, The doctor informs me about the various treatment options for my condition
	15, The doctor explains the advantages and disadvantages of different treatment options
	16, The doctor takes my opinions into account when making medical decisions
	11, The doctor provides active treatment and honestly informs me about my condition
	6, The doctor asks about any difficulties I encounter while receiving treatment
	7, The doctor's treatment makes me less concerned about my health condition
F2	4, Following the doctor's treatment plan can improve my health condition
	3, The doctor advises me to monitor symptom changes to seek timely medical attention
	5, The diet and lifestyle recommended by the doctor contribute to my recovery
	1, The doctor explains the purpose and necessity of the examination to me

F1 includes items primarily related to the doctor's communication effectiveness, information provision, decision-making involvement, and emotional support during the diagnosis and treatment process, reflecting the patient's perception of medical information transparency and humanistic care. Items 12, 14, and 15 directly reflect the doctor's communication effectiveness; items 8 and 10 reflect the emotional value provided by the doctor; items 16 and 6 indirectly reflect whether the doctor has decision-making consensus; and items 7, 13, and 11 reflect the doctor's treatment attitude. This dimension aligns with the “Patient-Centered Communication” (PCC) theory (39), which emphasizes that doctors should not only provide medical knowledge but also establish a strong trust relationship and interactive experience with patients during the medical process. F2 items focus more on the doctor's guidance of the patient's health behaviors and the promotion of medical adherence, specifically how the doctor encourages the patient to actively cooperate with treatment, monitor health changes, and practice health management. This dimension is related to the “Health Belief Model” (HBM) (40, 41), which posits that a patient's health behavior adherence is influenced by factors such as the perceived severity of the disease, health beliefs, and the doctor's advice. Studies have shown that doctors with better communication skills are more likely to inquire about patients' concerns, encourage exercise, and help set goals (42). These two dimensions jointly form a tripartite model of doctor-patient communication quality—emotional, cognitive, and behavioral—addressing the limitations of existing scales that tend to focus on a single aspect of communication behavior or attitude. The DPCQ developed in this study integrates concepts from PCC and HBM, balancing emotional support, information exchange, and behavioral guidance. It is designed to comprehensively assess the multidimensional communication experiences of patients within the context of the Chinese healthcare system.

In China, as healthcare reform continues to advance and public health awareness increases, patients are placing growing importance on the quality of doctor-patient communication. However, high-quality communication remains difficult to achieve in practice due to longstanding issues such as information asymmetry between doctors and patients, short consultation times, limited medical personnel, and the frequent occurrence of doctor-patient conflicts. Respect, empathy, and effective communication are the core elements of patient-centered care (43). Items 8 and 13 reflect patients' dual expectations of both professional competence and humanistic attitudes from physicians, indicating their need for both technical and emotional reassurance in an increasingly complex healthcare system. Items 12, 14, 15, and 16 address themes such as information transparency and shared decision-making, highlighting patients' urgent demand for informed consent and collaborative decision-making when facing multiple treatment options. These findings suggest that patients are no longer content with being passive recipients of care but prefer to engage in interactive relationships with their doctors. Furthermore, Items 4, 5, 6, and 7 reflect physicians' roles in guiding and promoting patients' long-term health behaviors, which align with China's current national policies on chronic disease management and the “Healthy China 2030” strategy (44, 45). As socioeconomic development leads to a transformation in public health perceptions, physicians play an increasingly vital role in maintaining patients' health and improving their health literacy (8). By incorporating dimensions such as “lifestyle guidance” and “symptom monitoring reminders,” the DPCQ emphasizes the value of doctor-patient communication from a health promotion perspective.

## 6 Conclusion

Through the study, a DPCQ (Doctor-Patient Communication Quality) measurement tool was developed, consisting of 14 items across two factors. The overall model's reliability, validity, and discriminant validity results suggest that the measurement model has good explanatory power and adaptability, although some items still need optimization. A higher total score on the DPCQ indicates better doctor-patient communication quality, which could lead to improved doctor-patient relationships and better medical outcomes. The application of the DPCQ can quantify the doctor-patient communication quality from the patient's perspective, providing data support and technical assistance for medical institutions to improve patient satisfaction, enhance doctor-patient relationships, and promote the development of the healthcare industry.

However, the generalizability of the DPCQ is limited. For example, there are significant differences in the social culture and healthcare systems across countries, and this DPCQ may only be applicable in the context of China.

## Data Availability

The original contributions presented in the study are included in the article/Supplementary material, further inquiries can be directed to the corresponding authors.

## Supplementary material

Supplementary Data Sheet 1 Doctor-Patient Communication Quality Questionnaire.

Supplementary Data Sheet 2 Doctor-Patient Communication Quality Scale.

Supplementary Data Sheet 3 Chinese Version of the Doctor-Patient Communication Quality Scale.
