# Supplementary material for: Development of a measurement of doctor-patient communication quality scale
Source: Front Public Health. 2025 Aug 11;13:1606403. doi: 10.3389/fpubh.2025.1606403 (PMC12375671; doi:10.3389/fpubh.2025.1606403)
Supplement: Supplementary Data Sheet 1 — Doctor-Patient Communication Quality Questionnaire. [file Data_Sheet_1.pdf]

Survey on Doctor-Patient Communication Quality

Dear Sir/Madam,

Hello! This questionnaire aims to assess the current state of doctor-patient communication quality among the general public and provide insights for improving doctor-patient relationships. This study is conducted by Anhui Medical University, and completing the questionnaire will take approximately 10 minutes.

Please answer based on your true feelings and experiences. There are no right or wrong answers, and your responses will be used solely for academic research. We guarantee that your information will remain confidential and will never be disclosed at any time.

Your participation is of great significance to this study. We sincerely appreciate your support and cooperation.

— Social Risk Management and Decision-Making Research Team,
Anhui Medical University

Basic Information

1. What is your gender?
    A. Male
    B. Female
2. What is your ethnicity?
    A. Han
    B. Other
3. What is your age? ______ years old
4. What is your highest level of education?
    A. High school or below
    B. Junior college
    C. Bachelor's degree
    D. Postgraduate degree
5. Do you have a religious belief?
    A. Yes
    B. No
6. What is your marital status?
    A. Unmarried
    B. Married (currently with a spouse)
    C. Other
7. Do you have children?
    A. No
    B. Yes, one child
    C. Yes, two children
    D. Yes, three or more children
8. Where have you primarily lived in the past six months?
    A. Rural
    B. Town
    C. Urban

9. What is your occupation?
A. Medical staff
B. Civil servant
C. Employee in a company/institution
D. Other
10. What is your household's per capita monthly income?
A. ≤ 2,000 RMB
B. 2,001–5,000 RMB
C. 5,001–8,000 RMB
D. 8,001–10,000 RMB
E. ≥ 10,000 RMB

Please evaluate your perception of the doctor's behavior during the diagnosis and treatment process.

Please evaluate your perception of the doctor's behavior during the diagnosis and treatment process.

1. The doctor explains the purpose and necessity of the examination to me.
A. Strongly disagree
B. Disagree
C. Neutral
D. Agree
E. Strongly agree
2. If the doctor does not explain clearly and just asks me to follow instructions, I feel confused about the diagnosis and treatment process.
A. Strongly disagree
B. Disagree
C. Neutral
D. Agree
E. Strongly agree
3. The doctor advises me to monitor symptom changes to seek timely medical attention.
A. Strongly disagree
B. Disagree
C. Neutral
D. Agree
E. Strongly agree
4. Following the doctor's treatment plan can improve my health condition.
A. Strongly disagree
B. Disagree
C. Neutral
D. Agree
E. Strongly agree
5. The diet and lifestyle recommended by the doctor contribute to my recovery.
A. Strongly disagree
B. Disagree

C. Neutral
D. Agree
E. Strongly agree

6. The doctor asks about any difficulties I encounter while receiving treatment.
   A. Strongly disagree
   B. Disagree
   C. Neutral
   D. Agree
   E. Strongly agree
7. The doctor's treatment makes me less concerned about my health condition.
   A. Strongly disagree
   B. Disagree
   C. Neutral
   D. Agree
   E. Strongly agree
8. The doctor has a warm and friendly attitude toward me.
   A. Strongly disagree
   B. Disagree
   C. Neutral
   D. Agree
   E. Strongly agree
9. The doctor listens carefully to what I have to say.
   A. Strongly disagree
   B. Disagree
   C. Neutral
   D. Agree
   E. Strongly agree
10. The doctor cares about the details of my life.
    A. Strongly disagree
    B. Disagree
    C. Neutral
    D. Agree
    E. Strongly agree
11. The doctor provides active treatment and honestly informs me about my condition.
    A. Strongly disagree
    B. Disagree
    C. Neutral
    D. Agree
    E. Strongly agree
12. The doctor answers my questions thoroughly and clearly.
    A. Strongly disagree
    B. Disagree
    C. Neutral
    D. Agree

E. Strongly agree

13. The doctor carefully considers my condition when diagnosing it.
    A. Strongly disagree
    B. Disagree
    C. Neutral
    D. Agree
    E. Strongly agree
14. The doctor informs me about the various treatment options for my condition.
    A. Strongly disagree
    B. Disagree
    C. Neutral
    D. Agree
    E. Strongly agree
15. The doctor explains the advantages and disadvantages of different treatment options.
    A. Strongly disagree
    B. Disagree
    C. Neutral
    D. Agree
    E. Strongly agree
16. The doctor takes my opinions into account when making medical decisions.
    A. Strongly disagree
    B. Disagree
    C. Neutral
    D. Agree
    E. Strongly agree
