# Supplementary material for: Development of a measurement of doctor-patient communication quality scale
Source: Front Public Health. 2025 Aug 11;13:1606403. doi: 10.3389/fpubh.2025.1606403 (PMC12375671; doi:10.3389/fpubh.2025.1606403)
Supplement: Supplementary Data Sheet 2 — Doctor-Patient Communication Quality Scale. [file Data_Sheet_2.pdf]

Doctor-Patient Communication Quality Scale (DPCQ)

This scale uses a Likert 5-point scale for scoring. The scoring criteria are as follows:
Strongly Disagree: 1 point
Disagree: 2 points
Neutral: 3 points
Agree: 4 points
Strongly Agree: 5 points
Please select the option that best reflects your true feelings for each item.

**Items**	**1**	**2**	**3**	**4**	**5**
1.The doctor explains the purpose and necessity of the examination to me					
2. The doctor advises me to monitor symptom changes to seek timely medical attention					
3. Following the doctor's treatment plan can improve my health condition					
4. The diet and lifestyle recommended by the doctor contribute to my recovery					
5. The doctor asks about any difficulties I encounter while receiving treatment					
6. The doctor's treatment makes me less concerned about my health condition					
7. The doctor has a warm and friendly attitude					
8. The doctor cares about the details of my life					
9. The doctor provides active treatment and honestly informs me about my condition					
10. The doctor answers my questions thoroughly and					
11. The doctor carefully considers my condition when diagnosing it					
12. The doctor informs me about the various treatment options for my condition					
13. The doctor explains the advantages and disadvantages of different treatment options					
14. The doctor takes my opinions into account when making medical decisions					
