# Supplementary material for: Development of a measurement of doctor-patient communication quality scale
Source: Front Public Health. 2025 Aug 11;13:1606403. doi: 10.3389/fpubh.2025.1606403 (PMC12375671; doi:10.3389/fpubh.2025.1606403)
Supplement: Supplementary Data Sheet 3 — Chinese Version of the Doctor-Patient Communication Quality Scale. [file Data_Sheet_3.pdf]

医生-患者沟通质量量表（DPCQ）

采用**李克特五项量表**进行评分。每个问题的评分标准如下：
完全不认同 代表您的感受与题目描述完全不符，赋予 1 分。
比较不认同 代表您的感受与题目描述较少一致，赋予 2 分。
一般认同 代表您的感受与题目描述部分一致，赋予 3 分。
比较认同 代表您的感受与题目描述较为一致，赋予 4 分。
完全认同 代表您的感受与题目描述完全一致，赋予 5 分。
请根据您的真实感受，在每个题目后选择一个最符合的选项。

题项	1	2	3	4	5
1.医生会跟我说明检查的目的和必要性					
2.医生会要求我注意症状变化，以便及时就医					
3.遵循医生的诊疗方案可以改善我的健康状况					
4.医生提倡的饮食和生活方式有助于我康复					
5.医生会询问我在实际接受治疗时遇到的问题					
6. 医生的诊疗让我对自身健康状况不再那么担忧					
7. 医生对我的态度热情友好					
8. 医生会关心我的生活细节					
9. 医生对我进行积极的治疗，并如实告知我的病情					
10. 医生会充分回答我的问题并解释清楚					
11. 医生在诊断我的病情时深思熟虑					
12. 医生会告诉我所患疾病的各种治疗方法					
13. 医生会告诉我不同治疗方法的利弊					
14. 医生在做出医疗决策时会考虑我的意见					
